# In vitro synergistic antimicrobial activity of a combination of meropenem, colistin, tigecycline, rifampin, and ceftolozane/tazobactam against carbapenem-resistant *Acinetobacter baumannii*

**DOI:** 10.1038/s41598-022-11464-6

**Published:** 2022-05-09

**Authors:** Yong Guk Ju, Hak Joon Lee, Hong Soon Yim, Min-Goo Lee, Jang Wook Sohn, Young Kyung Yoon

**Affiliations:** 1 Institute of Emerging Infectious Diseases, Korea University College of Medicine, Seoul, Republic of Korea; 2 Department of Physiology, Korea University College of Medicine, Seoul, Republic of Korea; 3 Division of Infectious Diseases, Department of Internal Medicine, Korea University Anam Hospital, Korea University College of Medicine, 73, Inchon-ro, Seongbuk-gu, Seoul, 02841 Republic of Korea

**Keywords:** Drug discovery, Microbiology, Medical research

## Abstract

We investigated the in vitro activity of various antimicrobial combinations against carbapenem-resistant *Acinetobacter baumannii* (CRAB) isolates. The in vitro activity of six two-drug combinations against CRAB isolates collected from the blood samples of patients with bloodstream infection was evaluated using the checkerboard method and time-kill assay [0.5 ×, 1 ×, and 2 × minimum inhibitory concentration (MIC)] to identify potential synergistic and bactericidal two-drug combinations against CRAB isolates. The effects of meropenem, colistin, tigecycline, rifampin, and ceftolozane/tazobactam combinations were investigated. All 10 CRAB isolates in our study produced the OXA-58-type and OXA-23-type carbapenem-hydrolyzing oxacillinases. The colistin-ceftolozane/tazobactam combination showed synergistic effects in both the time-kill assay (using an antibiotic concentration of 1 × MIC) and the checkerboard method. It also showed bactericidal effects in the time-kill assay. For all 10 CRAB isolates, time-kill curves showed synergistic bactericidal activity of the colistin-ceftolozane/tazobactam combination at 0.5 × MIC. Overall, there was substantial discordance of synergistic activity between the checkerboard microdilution and time-kill assays (with a concordance of 31.7%). Our study demonstrated that two-drug combinations of colistin and ceftolozane/tazobactam could be useful treatment alternatives for CRAB infections. The effects of these antibiotic combinations should be evaluated using in vivo experimental models.

## Introduction

Carbapenem-resistant *Acinetobacter baumannii* (CRAB), a leading nosocomial pathogen, poses a global threat to public health^1^. This pathogen is resistant to most clinically available antibiotics, and other treatment options are extremely limited. As a result, infection-related mortality has increased^2–4^. Furthermore, the spread of CRAB in a hospital environment makes infection control difficult. These bacteria can colonize various body parts of hospitalized patients and survive for a long time on various surfaces in hospital facilities^5,6^.

In the Republic of Korea, the carbapenem resistance rate of *A. baumannii* isolated from patients hospitalized in intensive care units was 90%^7,8^. According to the Korean arm of the Global Antimicrobial Resistance Surveillance System, CRAB is the most common multidrug-resistant pathogen causing bloodstream infections in intensive care units, with an incidence of 6.3 cases per 10,000 patient-days^9^.

The World Health Organization has designated CRAB as a pathogen of critical priority in the global priority list of multidrug-resistant bacteria and has urged the development of new antibiotics^10^. Despite relentless attempts to improve therapeutic approaches, there is no new promising antibiotic that can suitably control CRAB infections^11^. Currently, only a few antibiotics of uncertain efficacy, such as colistin and tigecycline, are available for treating CRAB infections. The reduced antibiotic susceptibility of CRAB, unfavorable pharmacokinetic properties, unclear optimal dosing, and potential adverse effects are all barriers to the clinical use of existing drugs such as colistin and tigecycline.

Given the increasing multidrug-resistance rates and the lack of effective antibiotics, combination therapy should be considered as an alternative interim strategy for effectively managing CRAB infections. Antimicrobial combination therapy may broaden the spectrum of activity, minimize the development of antimicrobial resistance, and synergistically inactivate microorganisms. Proposed mechanisms for synergistic antibacterial effects include enhanced bioavailability, inhibitor suppression, sequential blockade, mutual stabilization, parallel pathway inhibition, and regulation modulation^12^. However, evaluating the in vitro activity of antimicrobial combinations in clinical microbiology laboratories is challenging, as the experimental process is labor-intensive, time-consuming, and requires specialized skills. Furthermore, several methods available to evaluate the in vitro activity of antimicrobial combinations for CRAB isolates do not always show consistent results^13–15^. Nevertheless, several studies have addressed the therapeutic potential of combination therapy against CRAB infections^16–20^.

The purpose of this study was to investigate the in vitro activity of antimicrobial combinations of meropenem, colistin, tigecycline, rifampin, and ceftolozane/tazobactam against CRAB isolates producing OXA-23 carbapenemases.

## Methods

### Study population

A total of 158 clinical isolates of *A. baumannii* were collected from nonduplicate patients with CRAB bacteremia in a 1,048-bed tertiary care hospital in Seoul, Republic of Korea from April 2018 to January 2020. Ten clinical isolates of CRAB exhibiting resistance to imipenem, meropenem, and ertapenem were randomly selected for the study^21^.

The study protocol was approved prior to study initiation by the Institutional Review Board of Korea University Anam Hospital [No. 2020AN0157]. The study was performed in accordance with the ethical principles outlined in the Declaration of Helsinki. Informed consent was obtained from all subjects involved in this study.

### Bacterial isolates and antimicrobial susceptibility testing

The identification and antimicrobial susceptibility testing of *A. baumannii* strains were initially performed using the MicroScan Pos Combo Panel Type 6 automated system (Baxter Diagnostics, West Sacramento, CA, USA) in a clinical microbiology laboratory. The identity of the *A. baumannii* strains was confirmed using matrix-assisted laser desorption/ionization-time of flight mass spectrometry (Bruker Daltonics, Bremen, Germany).

The minimum inhibitory concentration (MIC) was determined for 15 antimicrobial agents using the broth microdilution method. Cation-adjusted Mueller–Hinton II broth (CA-MHB) (Becton Dicknson & Co., Sparks, MD, USA) was used, and geometric twofold serial dilutions were performed according to the CLSI recommendations^21,22^ for the following antimicrobial agents: meropenem (Yuhan Co., Seoul, Korea), ertapenem (MSD-Chibret, France), colistin (SteriMax Inc., ON, Canada), amikacin (Shinpoong Co., Seoul, Korea), tigecycline (Pfizer Pharmaceuticals, Seoul, Korea), ceftolozane/tazobactam (Wyeth, Madison, NJ, USA), piperacillin/tazobactam (Chong Kun Dang Pharmaceutical Co., Seoul, Korea), ampicillin/sulbactam (Keun-Hwa Pharmaceutical Co., Seoul, Korea), ceftazidime (Hanmi Co., Seoul, Korea), cefepime (Boryung Co., Seoul, Korea), aztreonam (Crystal Lifesciences, Cheonju, Korea), minocycline (SK Chemical Co., Seongnam, Korea), fosfomycin (Pharmbio Co., Chungju, Korea), rifampin (Yuhan Co., Seoul, Korea), and ciprofloxacin (Bayer AG, Leverkusen, Germany). Independent experiments were performed in duplicate. The MIC results were interpreted according to the CLSI breakpoint criteria^21^. Because the CLSI guidelines did not include breakpoint criteria for tigecycline, we used the criteria of the United States Food and Drug Administration for Enterobacteriaceae for tigecycline (susceptibility, 2 mg/L; resistance, 8 mg/L)^23^. For interpretation of colistin and rifampin susceptibilities, we used the breakpoints proposed by Gales et al. (resistance, 4 mg/L) and Hogg et al. (resistance, 2 mg/L), respectively^24,25^. *Pseudomonas aeruginosa* (ATCC 27853) was used as a quality control isolate.

All CRAB isolates from blood cultures were stored in brain heart infusion broth (Becton Dickinson & Co., Sparks, MD, USA) containing 20% glycerol and frozen at − 70 °C until February 2020. Then the isolates were thawed, and primary and secondary cultures were inoculated in 5% sheep blood agar for the experiments.

### Detection of carbapenem resistance determinants

For all CRAB isolates, Ambler class B metallo-β-lactamase genes (*bla*_IMP-1_, *bla*_IMP-2_, *bla*_VIM-1_, and *bla*_VIM-2_) and Ambler class D OXA-type carbapenemases-encoding genes (*bla*_oxa-23_, *bla*_oxa-24_, *bla*_oxa-51_, and *bla*_oxa-58_) were detected using simplex or multiplex polymerase chain reaction (PCR) techniques. The primers used in this study are shown in Table 1.

**Table 1:** Polymerase chain reaction (PCR) primers, conditions, and product size for the detection of carbapenemases among *Acinetobacter baumannii* strains.

Target gene	Primers (5′ → 3′)	Annealing temperature for multiplex PCR (°C)	Product size (bp)
*bla*_IMP-1_	ACCGCAGCAGAGTCTTTGCC ACAACCAGTTTTGCCTTACC	55	587
*bla*_IMP-2_	GTTTTATGTGTATGCTTCC AGCCTGTTCCCATGTAC	55	678
*bla*_VIM-1_	GGGAGCCGAGTGGTGAGT GGCACAACCACCGTATAG	55	519
*bla*_VIM-2_	ATGTTCAAACTTTTGAGTAAG CTACTCAACGACTGAGCG	55	801
*bla*_oxa-23_	GATCGGATTGGAGAACCAGA ATTTCTGACCGCATTTCCAT	52	501
*bla*_oxa-24_	GGTTAGTTGGCCCCCTTAAA AGTTGAGCGAAAAGGGGATT	52	246
*bla*_oxa-51_	TAATGCTTTGATCGGCCTTG TGGATTGCACTTCATCTTGG	52	353
*bla*_oxa-58_	AAGTATTGGGGCTTGTGCTG CCCCTCTGCGCTCTACATAC	52	599

Each reaction mixture (20 µL) contained 1 µL of genomic DNA, 10 pmol of each primer, 1 U of *Taq* DNA polymerase, 0.25 mM dNTP, 10 mM Tris–HCl (pH 9.0), 40 mM KCl, and 1.5 mM MgCl_2_. PCR conditions for amplifying the Ambler class B metallo-β-lactamase genes were as follows: 94 °C for 5 min, followed by 30 cycles of 94 °C for 45 s, annealing at the temperature specified for each set of primers for 1 min, and 72 °C for 1 min, followed by a final extension at 72 °C for 7 min (Table 1). PCR conditions for amplifying the Ambler class D OXA-type carbapenemase-encoding genes were as follows: 94 °C for 5 min, and then, 30 cycles of 94 °C for 30 s, annealing at the temperature specified for each set of primers for 40 s and 72 °C for 50 s, followed by a final extension at 72 °C for 7 min (Table 1).

### Checkerboard assays for synergy testing

The synergistic activities of various two-drug combinations against the 10 CRAB isolates were evaluated using the checkerboard assay, which was conducted in 96-well microtiter plates (Corning Inc., Kennebunk, ME, USA). The 10 isolates selected for in vitro synergy tests were chosen randomly without any specific criteria. Ceftolozane/tazobactam was recently approved, and data relating to it is limited. In addition to ceftolozane/tazobactam, we selected antibiotics for in vitro synergic tests based on a review of the literature on antibiotic combinations likely to be especially powerful in a clinical environment. In brief, panels of 96-well microtiter plates were prepared based on the MIC of each antibiotic, as determined using broth microdilution. Dilution intervals were determined to be 2–32 times higher than and 1/8–1/64 times lower than the MIC values obtained from the preliminary analysis. The antibiotic stock solutions were diluted with CA-MHB, and the concentrations of the upper left parts of the plates were set to 0. The plate rows contained 100 µL of the two-fold serial dilutions of the first antibiotic in each well, and the plate columns contained 100 µL of the two-fold serial dilutions of the second antibiotic. Test concentration ranges for each antibiotic in the experimental combinations were as follows: colistin, 0–128 mg/L; meropenem, 0–128 mg/L; tigecycline, 0–8 mg/L; ceftolozane/tazobactam, 0–128 mg/L; and rifampin, 0–256 mg/L.

The *A. baumannii* inoculum consisted of two-fold diluted 0.5 McFarland turbidity standard (100 µL) prepared using CA-MHB. The final inoculum concentration was 5 × 10^5^ CFU/mL in each well. Except for the sterility control well, all the wells were inoculated and then incubated at 37 °C for 18–20 h. At the stationary phase, the wells were diluted to an OD of 600 nm, as measured with an absorbance microplate reader (SpectraMax Plus 384, Molecular Devices, Inc). The MIC was visually determined by identifying the wells in the microtiter plate that had the lowest drug concentrations and no visible growth. The fractional inhibitory concentration index (FICI) was calculated using the formula below.

FICI = [(MIC of drug A in combination)/(MIC of drug A alone)] + [(MIC of drug B in combination)/(MIC of drug B alone)].

Interpretation of the FICI was as follows: FICI ≤ 0.5, synergistic; 0.5 < FICI ≤ 1, additive; 1 < FICI ≤ 4, indifferent; and FICI > 4, antagonistic^26^.

### Time-kill assay for synergy testing

In addition to the checkerboard assay, time-kill assays were conducted using the 10 *A. baumannii* isolates. In brief, tubes containing freshly prepared CA-MH broth supplemented with antibiotics, alone and in combination, were inoculated with CRAB isolates at a concentration of 10^4^ CFU/mL. The final volume of the suspensions in the tubes was 10 mL (in each tube); the tubes were incubated at 37 °C in a shaking incubator (200 rpm) in ambient air.

Then, 100-µL aliquots were obtained from each tube at 0, 2, 4, 8, 12, and 24 h of incubation and serially diluted in saline for the determination of viable counts. Diluted samples (10 µL) were plated on CA-MHA plates using a spreader (SPL Life Science Co.) and incubated at 37 °C for 18–24 h, and then, the number of colonies was counted. The antibiotic carry-over effect was minimized by washing the aliquots in sterile phosphate-buffered saline (PBS) and then centrifuging them for 5 min at 1,300 rpm before a tenfold serial dilution in sterile PBS. The initial bacterial density of the original sample was calculated based on the dilution factor. The lower limit of detection for the colony counts was 2 log_10_ CFU/mL. The concentrations of the antibiotics used were 0.5 × MIC, 1 × MIC, and 2 × MIC alone or in combination.

The bactericidal activity of single antibiotics or combinations was defined as a decrease of ≥ 3 log_10_ in 24 h compared with the number of viable cells at the initial time point^27^. A synergistic effect was defined as a decrease of ≥ 2 log_10_ CFU/mL within 24 h when the antibiotics in combinations were compared with the most active individual drug at different time points. An increase of > 2 log_10_ was considered to indicate antagonism. Indifference was defined as any outcome that did not meet the criteria for either synergy or antagonism^28^.

### Ethics committee approval

The study protocol was approved before study initiation by the Institutional Review Board of Korea University Anam Hospital [No. 2020AN0157].

## Results

### Characteristics of *A. baumannii* clinical isolates

The clinical isolates involved in this study are listed in Table 2. Out of 10 patients with CRAB bacteremia, 50% had in-hospital mortality. All 10 CRAB isolates carried the OXA-58-type and OXA-23-type carbapenem-hydrolyzing oxacillinases but did not harbor class B metallo-carbapenemases or other class D carbapenemases. Ten clinical isolates were resistant to meropenem, with MICs of 64 mg/L. Among them, the susceptibility rate associated with each antibiotic was as follows: tigecycline 90%, minocycline 100%, rifampin 30%, colistin 10%, ceftolozane/tazobactam 0%, ciprofloxacin 0%, fosfomycin 0%, aztreonam 0%, ceftazidime 0%, ampicillin/sulbactam 0%, piperacillin/tazobactam 0%, and amikacin 0% (Table 2).

**Table 2:** Clinical and microbiological characteristics of *Acinetobacter baumannii* isolates.

Isolate no	Patient information				Carbapenemase genes		MICs of antimicrobial agents (mg/L)*												
	Age	Sex	Clinical specimen	In-hospital mortality	*bla*_oxa-51_	*bla*_oxa-23_	Me	C	T	C/T	R	Cip	F	Mi	Az	Cef	A/S	P/T	Am
ATCC27853	−	−	−	−	−	−	0.25	2	4	0.5	**64**	0.125	4	**16**	8	2	**> 128**	4	1
CRAB 19	90	M	Blood	No	+	+	**64**	2	0.5	**128**	4	**16**	**> 128**	0.25	**128**	**> 128**	**64**	**> 128**	**128**
CRAB 32	66	F	Blood	Yes	+	+	**64**	**8**	0.5	**16**	4	**32**	**128**	0.25	**64**	**128**	**64**	**> 128**	**> 128**
CRAB 33	95	F	Blood	Yes	+	+	**64**	**8**	0.5	**32**	4	**64**	**128**	0.25	**64**	**128**	**64**	**> 128**	**> 128**
CRAB 34	81	F	Blood	No	+	+	**64**	**8**	0.5	**64**	**> 128**	**64**	**128**	0.25	**128**	**> 128**	**64**	**> 128**	**> 128**
CRAB 35	75	F	Blood	No	+	+	**64**	**4**	0.5	**64**	**> 128**	**64**	**128**	0.25	**128**	**> 128**	**64**	**> 128**	**> 128**
CRAB 36	57	F	Blood	No	+	+	**64**	**8**	0.5	**64**	**> 128**	**128**	**128**	0.25	**128**	**> 128**	**64**	**> 128**	**> 128**
CRAB 37	66	M	Blood	No	+	+	**64**	**8**	0.5	**32**	**> 128**	**64**	**128**	0.25	**128**	**> 128**	**64**	**> 128**	**> 128**
CRAB 38	81	F	Blood	Yes	+	+	**64**	**8**	0.5	**64**	**> 128**	**64**	**128**	0.25	**128**	**> 128**	**64**	**> 128**	**> 128**
CRAB 39	68	M	Blood	Yes	+	+	**64**	**4**	1	**32**	**> 128**	**64**	**128**	0.25	**128**	**> 128**	**64**	**> 128**	**> 128**
CRAB 40	89	M	Blood	Yes	+	+	**64**	**8**	**4**	**32**	**> 128**	**128**	**64**	0.5	**64**	**> 128**	**64**	**> 128**	**> 128**

*Am* amikacin, *A/S* ampicillin/sulbactam, *Az* aztreonam, *Cef* ceftazidime, *CRAB* carbapenem-resistant *Acinetobacter baumannii*, *Cip* ciprofloxacin, *C* colistin, *C/T* ceftolozane/tazobactam, *F* female, *F* fosfomycin, *M* male, *Me* meropenem, *Mi* minocycline, *MIC* minimum inhibitory concentration, *P/T* piperacillin-tazobactam, *R* rifampin, *T* tigecycline.

*Bold values, non-susceptible; white cell, susceptible.

### Checkerboard assay against *A. baumannii* clinical isolates

The results of the checkerboard assay used to measure the in vitro synergism and MIC values of the individual antibiotic combinations against the 10 CRAB isolates are summarized in Table 3. The checkerboard assay showed that in vitro synergistic activity (∑FICI ≤ 0.5) against CRAB isolates was the highest for the meropenem-tigecycline combination (90%), followed by the meropenem-ceftolozane/tazobactam (70%), meropenem-rifampin (70%), colistin-ceftolozane/tazobactam (60%), colistin-tigecycline (30%), and meropenem-colistin (30%) combinations (Table 3). All combinations displayed synergism to a certain extent.

**Table 3:** Results of checkerboard assay for two-drug combinations against clinical isolates of *Acinetobacter baumannii.*

Clinical isolates	MIC of a single agent (mg/L)		MIC in combination (mg/L)		FICI	Result of checkerboard assay
	Drug A	Drug B	Drug A	Drug B		
**Meropenem (A) + colistin (B)**						
CRAB 19	64	2	4	0.25	0.19	Synergistic
CRAB 32	64	8	16	0.5	0.31	Synergistic
CRAB 33	64	8	0.125	0.25	0.03	Synergistic
CRAB 34	64	8	32	0.25	0.53	Additive
CRAB 35	64	8	32	0.25	0.56	Additive
CRAB 36	64	8	32	0.5	0.56	Additive
CRAB 37	64	8	32	0.25	0.53	Additive
CRAB 38	64	8	32	0.25	0.53	Additive
CRAB 39	64	4	32	0.25	0.56	Additive
CRAB 40	64	8	32	0.25	0.53	Additive
**Meropenem (A) + tigecycline (B)**						
CRAB 19	64	0.5	0.125	0.03	0.06	Synergistic
CRAB 32	64	0.5	8	0.125	0.38	Synergistic
CRAB 33	64	0.5	32	0.5	1.5	Indifferent
CRAB 34	64	0.5	16	0.125	0.5	Synergistic
CRAB 35	64	0.5	16	0.125	0.5	Synergistic
CRAB 36	64	0.5	0.125	0.125	0.25	Synergistic
CRAB 37	64	0.5	0.125	0.125	0.25	Synergistic
CRAB 38	64	0.5	16	0.125	0.5	Synergistic
CRAB 39	64	1	16	0.125	0.38	Synergistic
CRAB 40	64	4	8	0.125	0.16	Synergistic
**Meropenem (A) + rifampin (B)**						
CRAB 19	64	4	32	0.25	0.56	Additive
CRAB 32	64	4	32	4	1.5	Indifferent
CRAB 33	64	4	16	2	0.75	Additive
CRAB 34	64	256	4	8	0.09	Synergistic
CRAB 35	64	256	16	4	0.27	Synergistic
CRAB 36	64	256	16	8	0.28	Synergistic
CRAB 37	64	256	16	4	0.27	Synergistic
CRAB 38	64	256	16	4	0.27	Synergistic
CRAB 39	64	256	16	4	0.27	Synergistic
CRAB 40	64	256	8	8	0.16	Synergistic
**Meropenem (A) + ceftolozane/tazobactam (B)**						
CRAB 19	64	128	32	16	0.63	Additive
CRAB 32	64	16	8	4	0.38	Synergistic
CRAB 33	64	32	8	16	0.63	Additive
CRAB 34	64	64	8	4	0.19	Synergistic
CRAB 35	64	64	2	2	0.06	Synergistic
CRAB 36	64	64	32	32	1	Additive
CRAB 37	64	32	4	2	0.13	Synergistic
CRAB 38	64	64	16	2	0.28	Synergistic
CRAB 39	64	32	2	4	0.16	Synergistic
CRAB 40	64	32	8	8	0.38	Synergistic
**Colistin (A) + tigecycline (B)**						
CRAB 19	2	0.5	0.006	0.03	0.06	Synergistic
CRAB 32	8	0.5	4	0.25	1	Additive
CRAB 33	8	0.5	0.5	0.25	0.56	Additive
CRAB 34	8	0.5	8	0.5	2	Indifferent
CRAB 35	8	0.5	4	0.25	1.5	Indifferent
CRAB 36	8	0.5	2	0.125	0.5	Synergistic
CRAB 37	8	0.5	8	0.5	2	Indifferent
CRAB 38	8	0.5	8	0.5	2	Indifferent
CRAB 39	4	1	4	1	2	Indifferent
CRAB 40	8	4	0.125	1	0.27	Synergistic
**Colistin (A) + ceftolozane/tazobactam (B)**						
CRAB 19	2	128	2	128	2	Indifferent
CRAB 32	8	16	4	8	1	Additive
CRAB 33	8	32	0.125	2	0.08	Synergistic
CRAB 34	8	64	0.5	8	0.19	Synergistic
CRAB 35	8	64	0.25	16	0.31	Synergistic
CRAB 36	8	64	8	64	2	Indifferent
CRAB 37	8	32	1	16	0.63	Additive
CRAB 38	8	64	2	2	0.28	Synergistic
CRAB 39	4	32	0.25	4	0.19	Synergistic
CRAB 40	8	32	0.125	8	0.27	Synergistic

*CRAB* carbapenem-resistant *Acinetobacter baumannii*, *FICI* fractional inhibitory concentration index, *MIC* minimum inhibitory concentration.

Notably, we did not observe antagonistic interactions in our study. The MICs of the antibiotic combinations were lower than the MICs of the antibiotics used by themselves.

### Time-kill assay against *A. baumannii* clinical isolates

All 10 CRAB isolates were also evaluated using the time-kill assay. When evaluating the bactericidal effects of antibiotic monotherapy on the 10 CRAB isolates, antibiotic concentrations of 0.5 × MIC, 1 × MIC, and 2 × MIC were used. Bactericidal activity was noted for meropenem (0.5 × MIC, 0%, 0/10; 1 × MIC, 40%, 4/10; 2 × MIC, 70%, 7/10), ceftolozane/tazobactam (0.5 × MIC, 0%, 0/10; 1 × MIC, 0%, 0/10; 2 × MIC, 70%, 7/10), colistin (0.5 × MIC, 0%, 0/10; 1 × MIC, 50%, 5/10; 2 × MIC, 70%, 7/10), rifampin (0.5 × MIC, 0%, 0/10; 1 × MIC, 10%, 1/10; 2 × MIC, 40%, 4/10), and tigecycline (0.5 × MIC, 10%, 1/10; 1 × MIC, 10%, 1/10; 2 × MIC, 40%, 4/10) at 24 h (Supplementary file).

For the time-kill assay performed using an antibiotic concentration of 1 × MIC, in vitro synergistic and bactericidal effects against the 10 CRAB isolates were most frequently observed for the meropenem-colistin (40% and 100%, respectively), colistin-ceftolozane/tazobactam (50% and 100%, respectively), and colistin-tigecycline (40% and 100%, respectively) combinations. These effects were seen less often for the meropenem-tigecycline (50% and 50%, respectively), meropenem-ceftolozane/tazobactam (50% and 50%, respectively), and meropenem-rifampin (20% and 30%, respectively) combinations at 24 h (Table 4).

**Table 4:** Results of time-kill assay and bactericidal activity of two-drug combinations (1 × MIC) against each *Acinetobacter baumannii* isolate.

Isolate	Time-kill assay						Bactericidal activity (Timepoint, hours; 2/4/6/8/12/24)					
	Me + T	Me + C	Me + R	Me + C/T	C + T	C + C/T	Me + T	Me + C	Me + R	Me + C/T	C + T	C + C/T
CRAB 19	I	I	I	I	I	I	N/N/N/N/B	N/N/B/B/B	N/N/B/B/B	N/N/N/B/B	N/B/B/B/B	N/N/B/B/B
CRAB 32	I	Syn	I	Syn	I	Syn	N/N/N/B/B	N/N/B/B/B	N/N/N/N/N	N/N/N/N/B	N/N/N/B/B	N/N/B/B/B
CRAB 33	Syn	Syn	I	Syn	Syn	Syn	N/N/N/N/N	N/N/N/B/B	N/N/N/N/N	N/N/N/N/N	N/N/N/N/B	N/N/N/B/B
CRAB 34	Syn	I	Syn	Syn	I	I	N/N/N/N/B	N/N/N/B/B	N/N/N/B/B	N/N/N/N/B	N/B/B/B/B	B/N/B/B/B
CRAB 35	Syn	Syn	I	I	Syn	Syn	N/N/N/N/N	N/N/N/B/B	N/N/N/N/N	N/N/N/N/N	N/N/N/N/B	N/N/N/B/B
CRAB 36	Syn	I	I	Syn	I	I	N/N/N/B/B	N/N/N/B/B	N/N/N/N/N	N/N/N/N/B	N/N/B/B/B	N/N/N/B/B
CRAB 37	Syn	Syn	Syn	Syn	Syn	Syn	N/N/N/B/B	N/N/N/B/B	N/N/N/B/B	N/N/N/N/B	N/B/B/B/B	N/N/N/B/B
CRAB 38	An	I	An	An	I	I	N/N/N/N/N	N/N/N/B/B	N/N/N/N/N	N/N/N/N/N	N/N/N/N/B	N/N/N/B/B
CRAB 39	An	I	An	An	Syn	Syn	N/N/N/N/N	N/N/N/B/B	N/N/N/N/N	N/N/N/N/N	N/N/N/B/B	N/N/N/B/B
CRAB 40	I	I	An	An	I	I	N/N/N/N/N	N/N/N/B/B	N/N/N/N/N	N/N/N/N/N	N/N/N/B/B	N/N/N/B/B

*An* antagonism, *B* bactericidal, *C* colistin, *C/T* ceftolozane/tazobactam, *I* indifference, *Me* meropenem, *N* non-bactericidal, *R* rifampin, *Syn* synergy, *T* tigecycline, *MIC* minimum inhibitory concentration.

**Figure 1:**
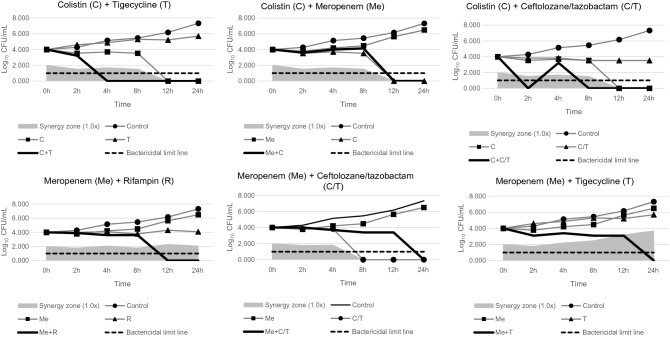
Time-kill curves of *Acinetobacter baumannii* isolates (CRAB 34) at 1 × minimum inhibitory concentration (MIC) for six combinations of antibiotics.

For the colistin-ceftolozane/tazobactam combination (both at levels of 1 × MIC), the 10 CRAB isolates yielded synergy rates of 60% after 12 h and 50% after 24 h (Table 5). For the meropenem-colistin combination (at 1 × MIC), the 10 CRAB isolates showed synergistic rates of 50% in 12 h and 40% in 24 h, respectively (Table 5). For the meropenem-ceftolozane/tazobactam combination (at 1 × MIC), the 10 CRAB isolates showed synergistic rates of 20% within 12 h and 50% within 24 h (Table 5). For the meropenem-tigecycline combination (at 1 × MIC), the 10 CRAB isolates showed synergistic rates of 40% within 12 h and 50% within 24 h (Table 5). However, the meropenem-rifampin combinations (at 1 × MIC) showed synergistic rates of only 20% at 8 h, 12 h, and 24 h. The colistin-tigecycline combinations (at 1 × MIC) also showed synergic rates of at most 40% within 24 h (Table 5). Overall, doubling the antibiotic concentration did not improve synergy rates for six combinations of antibiotics (Table 5). The synergistic inhibitory activity for the other antibiotic combinations was sustained for 24 h, except for the CRAB 34 isolates. These isolates demonstrated regrowth at 4 h for the colistin-ceftolozane/tazobactam combination (both at a concentration of 1 × MIC) (Fig. 1).

**Table 5:** Results of time-kill assays and the bactericidal effect of two-drug combinations against 10 *Acinetobacter baumannii* isolates.

	Bactericidal effect (A)						Synergy effect (B)						Both A and B					
	0 h	2 h (%)	4 h (%)	8 h (%)	12 h (%)	24 h (%)	0 h	2 h (%)	4 h (%)	8 h (%)	12 h (%)	24 h (%)	0 h	2 h (%)	4 h (%)	8 h (%)	12 h (%)	24 h (%)
**Me + T**																		
0.5x						20					20	20						10
1.0x					30	50					40	50					20	30
2.0x		10	10	10	40	100		10	10		10	30		10	10		10	30
**Me + C**																		
0.5x					10	20					10	40					10	20
1.0x				20	100	100				20	50	40				20	50	40
2.0x				30	100	100				10	20	20				10	20	20
**Me + R**																		
0.5x												10						
1.0x				10	30	30				20	20	20				10	20	20
2.0x			10	20	30	90			10	10		20			10	10		20
**Me + C/T**																		
0.5x						10					10	30						10
1.0x					10	50				10	20	50					10	40
2.0x				10	50	70				10	10					10	10	
**C + T**																		
0.5x				30	60	100				60	70	90				30	60	90
1.0x			30	40	70	100			30	40	40	40			30	40	30	40
2.0x		10	20	70	100	100		10		30	20	20		10		20	20	20
**C + C/T**																		
0.5x				20	50	100		10	10	50	80	100				20	50	100
1.0x		10		30	100	100		20	10	40	60	50		10		30	60	50
2.0x			40	50	100	100			20	10	30	30			20	10	30	30

The percentage values indicate the ratio of isolates showing bactericidal or synergistic effects among all 10 CRAB isolates.

*C* colistin, *C/T* ceftolozane/tazobactam, *Me* meropenem, *R*, rifampin, *T* tigecycline.

At concentrations of 1 × and 2 × MIC, combinations of colistin-ceftolozane/tazobactam (1 × MIC, 100%, 10/10; 2 × MIC, 100%, 10/10), colistin-tigecycline (1 × MIC, 100%, 10/10; 2 × MIC, 100%, 10/10), and meropenem-colistin (1 × MIC, 100%, 10/10; 2 × MIC, 100%, 10/10) usually showed bactericidal activity against the 10 CRAB isolates (Table 5).

In the time-kill assay tests that used antibiotic concentrations of 1 × MIC, the combinations that showed both bactericidal activity and a synergistic effect at 24 h were as follows: colistin-ceftolozane/tazobactam (50%), colistin-tigecycline (40%), meropenem-colistin (40%), meropenem-ceftolozane/tazobactam (40%), meropenem-tigecycline (30%), and meropenem-rifampin (20%) (Table 6). For over half of the 10 clinical isolates, the antibiotic combinations that showed bactericidal activity and a synergistic effect at the same time was colistin-ceftolozane/tazobactam (60% and 50% at 12 h and 24 h after inoculation, respectively) (Table 5).

**Table 6:** Comparison of checkerboard assays and time-kill assays associated with the bactericidal activity of two-drug combinations (1 × MIC) against each *Acinetobacter baumannii* isolate.

Isolate	Checkerboard assay results						Time-kill assay results					
	Me + T	Me + C	Me + R	Me + C/T	C + T	C + C/T	Me + T	Me + C	Me + R	Me + C/T	C + T	C + C/T
CRAB 19	Syn	Syn	Additive	Additive	Syn	I	I./B	I./B	I./B	I./B	I./B	**I./B**
CRAB 32	Syn	Syn	I	Syn	Additive	Additive	I./B	**Syn./B**	**I./N**	**Syn./B**	I./B	Syn./B
CRAB 33	I	Syn	Additive	Additive	Additive	Syn	Syn./N	**Syn./B**	I./N	Syn./N	Syn./B	**Syn./B**
CRAB 34	Syn	Additive	Syn	Syn	I	Syn	**Syn./B**	I./B	**Syn./B**	**Syn./B**	**I./B**	I./B
CRAB 35	Syn	Additive	Syn	Syn	I	Syn	**Syn./N**	Syn./B	I./N	I./N	Syn./B	**Syn./B**
CRAB 36	Syn	Additive	Syn	Additive	Syn	I	**Syn./B**	I./B	I./N	Syn./B	I./B	**I./B**
CRAB 37	Syn	Additive	Syn	Syn	I	Additive	**Syn./B**	Syn./B	**Syn./B**	**Syn./B**	Syn./B	Syn./B
CRAB 38	Syn	Additive	Syn	Syn	I	Syn	An./N	I./B	An./N	An./N	**I./B**	I./B
CRAB 39	Syn	Additive	Syn	Syn	I	Syn	An./N	I./B	An./N	An./N	Syn./B	**Syn./B**
CRAB 40	Syn	Additive	Syn	Syn	Syn	Syn	I./N	I./B	An./N	An./N	I./B	I./B

*An* antagonism, *B* bactericidal, *C* colistin, *C/T* ceftolozane/tazobactam, *I* indifference, *Me* meropenem, *N* non-bactericidal, *R* rifampin, *Syn* synergy, *T* tigecycline, *MIC* minimum inhibitory concentration.

*Bold values indicate that the results of the time-kill assay were consistent with the results of the checkerboard assays.

Regarding the synergistic effect of antibiotic combinations, 31.7% (19/60) of the results of the time-kill assay were consistent with the checkerboard results (Table 6). The combinations that simultaneously showed bactericidal and synergistic effects at 24 h in both the checkerboard and time-kill assays were as follows: colistin-ceftolozane/tazobactam (30%), meropenem-tigecycline (30%), meropenem-ceftolozane/tazobactam (30%), meropenem-colistin (20%), and meropenem-rifampin (20%) (Table 6).

**Figure 2:**
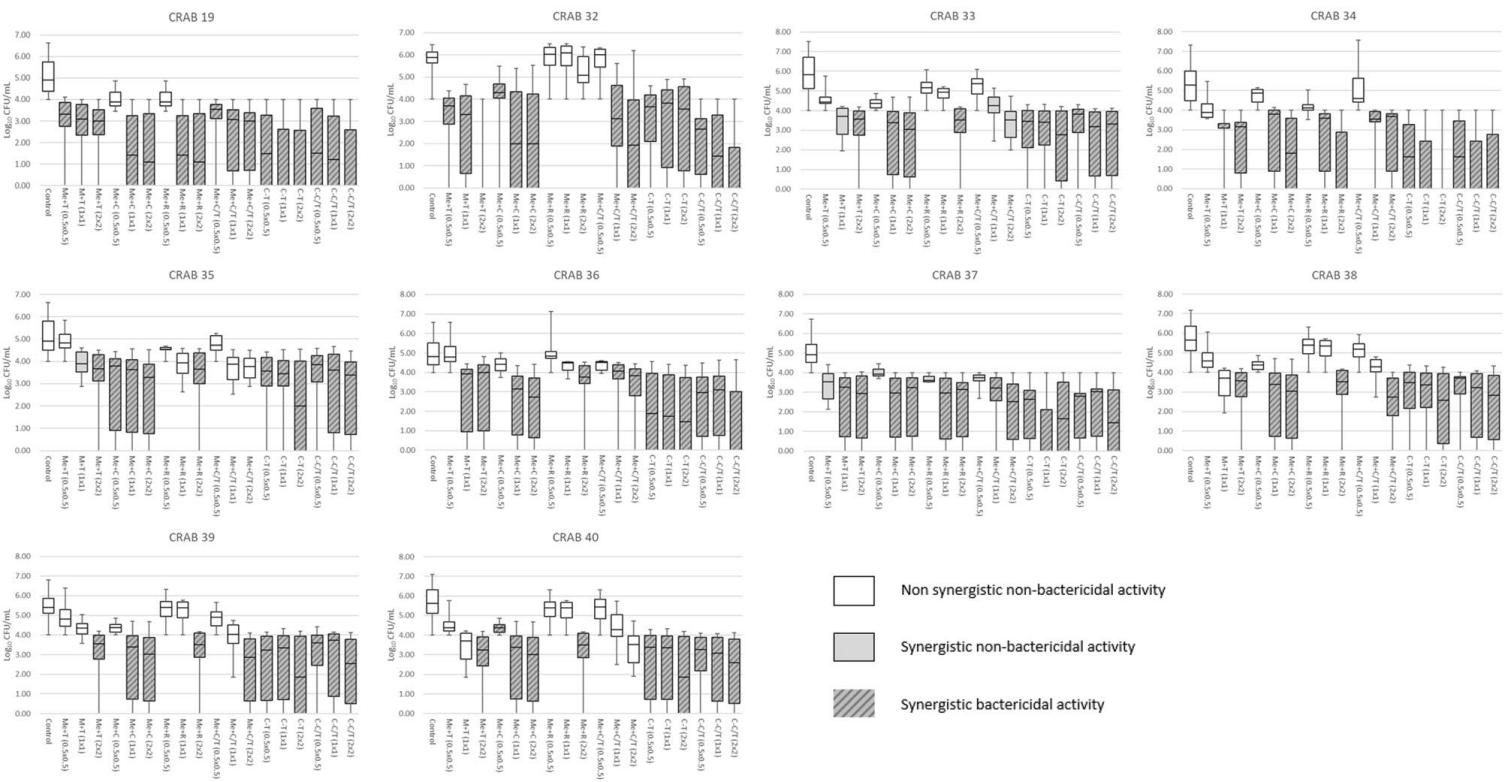
Time-kill curves of the 10 *Acinetobacter baumannii* isolates for six combinations of antibiotics with the different concentrations. C, colistin; C/T, ceftolozane/tazobactam; *Me* meropenem, *R* rifampin, *T* tigecycline.

Figure 2 shows the time-kill curves of six different combinations of antibiotics at concentrations of 0.5 ×, 1 ×, 2 × MIC against the CRAB isolates. In the time-kill assay, antagonism was observed for the meropenem-rifampin (30%), meropenem-ceftolozane/tazobactam (30%), and meropenem-tigecycline (20%) combinations (Table 4).

## Discussion

Our study was performed to determine which antibiotic combinations could be suitable options for treating CRAB infections. To the best of our knowledge, this is the first study to evaluate the in vitro synergistic activity of ceftolozane/tazobactam and other antibiotics against CRAB isolates. Our findings showed that the combination of colistin and ceftolozane/tazobactam had in vitro synergistic and bactericidal effects against OXA-23-type carbapenemase-producing CRAB isolates.

All 10 CRAB isolates in our study carried the OXA-58-type and OXA-23-type carbapenem-hydrolyzing oxacillinases. A majority of the CRAB isolates in the Republic of Korea have been shown to carry *bla*_oxa-23_^29^. All 10 CRAB isolates had an MIC of 64 mg/L for meropenem, and 90% of the CRAB isolates had an MIC of ≤ 1 mg/L for tigecycline. Particularly, MICs for colistin ranged from 2 to 8 mg/L. Notably, using an antibiotic concentration of 1 × MIC for the time-kill assay in our study failed to produce a stable bactericidal effect when antibiotic monotherapy was used against the 10 CRAB isolates. In this scenario, a combination antibiotic therapy could be the best way to treat CRAB infections. In addition, it is likely that any differences in susceptibility to each antibiotic would also alter the effects of any antibiotic combination.

The colistin-ceftolozane/tazobactam combination demonstrated a synergistic effect in both the time-kill assay and the checkerboard method, and it showed a bactericidal effect in the time-kill assay. In contrast, the meropenem-tigecycline, meropenem-ceftolozane/tazobactam, and meropenem-rifampin combinations showed antagonistic effects for some CRAB isolates in the time-kill assay.

Ceftolozane/tazobactam, a novel beta-lactam/beta-lactamase inhibitor, has demonstrated potent in vitro activity against *Pseudomonas aeruginosa*, including carbapenem-resistant isolates, except for class B carbapenemase producers. However, its activity against *A. baumannii* isolates is poor^30^. In our study, the susceptibility of CRAB isolates to ceftolozane/tazobactam when used as a single antibiotic was poor, with a MIC range of 16–128 mg/L. This finding is in accordance with previous results^31^. However, our findings from the time-kill assay (1 × MIC) identified the potential of the colistin and ceftolozane/tazobactam combination to induce synergistic interaction against all 10 CRAB isolates at 24 h. All 10 CRAB isolates in our study carried Ambler class D carbapenemases. Further studies are needed to assess the antimicrobial effect of regimens containing ceftolozane/tazobactam on CRAB isolates that exhibit different mechanisms of carbapenem resistance.

The time-kill curves for all 10 CRAB isolates showed that the colistin-ceftolozane/tazobactam combination exhibited significant synergistic bactericidal activity at 0.5 × MIC (Fig. 2). This finding has promising implications for using lower doses of colistin in treatment, thereby reducing its potential nephrotoxic effect.

We found that for the meropenem-tigecycline combination, in vitro synergistic activities took place in 90% and 50% of the 10 CRAB isolates in the checkerboard and time-kill assays, respectively. However, in vitro antagonistic activities were found in 20% of the 10 CRAB isolates in terms of the time-kill assay. A previous meta-analysis revealed a synergistic rate of 24.5% and 20.0% for CRAB isolates using the checkerboard (36 studies) and the time-kill (35 studies) methods, respectively^19^. It is possible that the high susceptibility rate (90%) of the CRAB isolates to tigecycline in our study contributed to these results. A recent clinical study reported that the colistin-tigecycline combination was associated with a higher mortality rate when the MIC of tigecycline was > 2 mg/L, which was achieved through the combination of a carbapenem and colistin^32^. Therefore, it is important to know the best way to select from existing antibiotic treatment regimens based on a strain’s antibiotic resistance phenotype (hospital antibiogram). This information can be used to achieve an improved clinical outcome. However, non-colistin-based combination regimens may play an important role in the treatment of CRAB infections, especially for those who are concerned about its nephrotoxic side effects and the emergence of hetero-resistance or resistance to colistin in CRAB isolates.

In our study, we incubated CRAB in the presence of antibiotics for 24 h, and the regrowth phenomenon was observed in the time-kill assay of the CRAB 34 isolate at 4 h after inoculation when the colistin-ceftolozane/tazobactam combination (with both at a concentration of 1 × MIC) was evaluated (Fig. 1). Although most of the time-kill analyses incubated bacteria in the presence of antibiotics for 24 h, incubation for 48 h may be better because it would allow improved detection of the regrowth phenomenon, owing to the selective amplification of the resistant subpopulation^33^. A previous study reported the regrowth phenomenon to be commonly detected in time-kill assays using colistin, despite the in vitro antimicrobial activity of colistin against the CRAB isolates^16^.

In our study, the meropenem-colistin combination showed 30% and 40% synergistic activities in the checkerboard and time-kill assays, respectively. The effectiveness of the meropenem-colistin combination remains an area of active research for the treatment of CRAB infections. A previous study that used time-kill assays to investigate the effects of different antibiotic combinations against 12 CRAB isolates at 5 × 10^5^ CFU/mL found that the meropenem-colistin combination was the most synergistic combination^34^. The checkerboard and time-kill assays showed synergistic effects of 60–73.3% and 60–96.1%, respectively^19,35–37^. In a previous meta-analysis, the synergistic rates shown by time-kill methods were significantly higher than those obtained using the checkerboard method, and our results are similar^20,38^. Overall, there is great discordance between the checkerboard microdilution and time-kill assays, and our study showed a concordance of 31.7%. In contrast, a previous meta-analysis showed a higher synergistic rate for CRAB isolates in a combination of meropenem and colistin, compared to a combination of imipenem and colistin^18,20^.

The present study has several limitations. First, the results do not apply to CRAB isolates that produce metallo-beta-lactamase. Notably, the CRAB isolates used in our study are highly resistant to meropenem, and they might have responded differently to the various antibiotic combinations if their MICs for meropenem were lower. Second, this study assessed the response of a small number of CRAB isolates using the checkerboard and time-kill assays. The MIC values of colistin in many of the CRAB isolates were remarkably high because only a few isolates were used to study the in vitro synergistic and bactericidal activities of the experimental antibiotic combinations. However, it is meaningful to collect isolates from patients with CRAB bacteremia in a clinical setting. Third, although antimicrobial susceptibility tests were performed in duplicate, this was not true for all the in vitro synergic tests. However, a strength of our in vitro synergy tests was that they tested a variety of antibacterial agents at different concentrations for their effect on 10 CRAB strains isolated in clinical practice. Finally, we acknowledge that in vitro studies do not always produce similar results in clinical practice; therefore, caution is required when applying these results in such a context.

## Conclusions

In conclusion, the present study demonstrated that the combination of colistin and ceftolozane/tazobactam may be a promising alternative to colistin alone for treating CRAB infections. However, the benefits of these antibiotic combinations should be validated using in vivo experimental models.

## Supplementary Information

Supplementary Information.

## Data Availability

The datasets generated and analyzed during the current study are available from the corresponding author on reasonable request.

## Abbreviations

Am: Amikacin
An: Antagonism
A/S: Ampicillin/sulbactam
Az: Aztreonam
B: Bactericidal
C: Colistin
CA-MHB: Cation-adjusted Mueller–Hinton II broth
Cef: Ceftazidime
Cip: Ciprofloxacin
CLSI: Clinical and Laboratory Standards Institute
CRAB: Carbapenem-resistant *Acinetobacter baumannii*
C/T: Ceftolozane/tazobactam
F: Female
FICI: Fractional inhibitory concentration index
F: Fosfomycin
I: Indifference
M: Male
Me: Meropenem
Mi: Minocycline
MIC: Minimum inhibitory concentration
N: Non-bactericidal
PCR: Polymerase chain reaction
P/T: Piperacillin-tazobactam
R: Rifampin
Syn: Synergy
T: Tigecycline
